# Imaging in disappearing colorectal liver metastases and their accuracy: a systematic review

**DOI:** 10.1186/s12957-020-02037-w

**Published:** 2020-10-08

**Authors:** Darius Barimani, Joonas H. Kauppila, Christian Sturesson, Ernesto Sparrelid

**Affiliations:** 1 Division of Surgery, Department of Clinical Science, Intervention, and Technology (CLINTEC), Center for Digestive Diseases, Karolinska University Hospital, Karolinska Institute, Stockholm, Sweden; 2 Surgery Research Unit, Oulu University Hospital, University of Oulu, Oulu, Finland

**Keywords:** Disappearing, Vanishing, Missing, Liver metastases, Colorectal metastases, Imaging, Complete response, Survival, Management

## Abstract

**Background:**

Approximately 30% of patients with colorectal cancer develop colorectal liver metastases (CRLM). CRLM that become undetectable by imaging after chemotherapy are called disappearing liver metastases (DLM). But a DLM is not necessarily equal to cure. An increasing incidence of patients with DLM provides surgeons with a difficult dilemma: to resect or to not resect the original sites of DLM? The aim of this review was to investigate to what extent a DLM equates a complete response (CR) and to compare outcomes.

**Methods:**

This review was conducted in accordance with the PRISMA guidelines and registered in Prospero (registration number CRD42017070441). Literature search was made in the PubMed and Embase databases. During the process of writing, PubMed was repeatedly searched and reference lists of included studies were screened for additional studies of interest for this review. Results were independently screened by two authors with the Covidence platform. Studies eligible for inclusion were those reporting outcomes of DLM in adult patients undergoing surgery following chemotherapy.

**Results:**

Fifteen studies were included with a total of 2955 patients with CRLM. They had 4742 CRLM altogether. Post-chemotherapy, patients presented with 1561 DLM. Patients with one or more DLM ranged from 7 to 48% (median 19%). Median DLM per patient was 3.4 (range 0.4–5.6). Patients were predominantly evaluated by contrast-enhanced computed tomography (CE-CT) before and after chemotherapy, with some exceptions and with addition of magnetic resonance imaging (MRI) in some studies. Intraoperative ultrasound (IOUS) was universally performed in all but two studies. If a DLM remained undetectable by IOUS, this DLM represented a CR in 24–96% (median 77.5%). Further, if a DLM on preoperative CE-CT remained undetectable by additional workup with MRI and CE-IOUS, this DLM was equal to a CR in 75–94% (median 89%). Patients with resected DLM had a longer disease-free survival compared to patients with DLM left in situ but statistically significant differences in overall survival could not be found.

**Conclusion:**

Combination of CE-CT, MRI, and IOUS showed promising results in accurately identifying DLM with CR. This suggests that leaving DLM in situ could be an alternative to surgical resection when a DLM remains undetectable by MRI and IOUS.

## Core tip

From a per-lesion perspective, this review shows that combining high-resolution gadoxetic acid-enhanced magnetic resonance imaging (EOB-MRI) with contrast-enhanced intraoperative ultrasound (CE-IOUS) to evaluate disappearing liver metastases (DLM) on preoperative contrast-enhanced computed tomography (CE-CT) can accurately identify cured DLM with high probability. These findings challenge the current dogma of mandatory resection of all DLM in favor of a watch and wait policy towards these carefully selected DLM. Whether this is safe from a per-patient perspective is subject to further research. Additionally, heterogeneity of future studies on DLM could be avoided by adhering to a more uniform nomenclature. This review calls for a prospective study comparing resection vs. no resection in patients with DLM.

## Introduction

Approximately 50% of all patients with colorectal cancer develop colorectal liver metastases (CRLM) [1]. Only 20–30% of the patients with CRLM are candidates for surgical resection upon diagnosis [2]. Conversion chemotherapy may be offered to patients with CRLM that are not candidates for resection. Patients with resectable CRLM may be offered neoadjuvant chemotherapy. Neoadjuvant treatment for CRLM involves the use of combinations of chemotherapeutic agents to improve prognosis [3]. Chemotherapy is often combined with biological agents to achieve resectability in initially unresectable CRLM.

When CRLM disappear on preoperative imaging following chemotherapy, they are known as disappearing liver metastases (DLM) or vanishing lesions. With advancements in chemotherapy and biological agents, the occurrence of DLM is becoming increasingly common [4]. Whether some of these DLM represent cure, i.e., a complete response (CR), is subject to speculation. Current management of DLM consists predominantly of an aggressive surgical approach, where resection of all the sites of the DLM is attempted. Disappeared liver metastases are left in the remnant liver only when complete resection of DLM would leave the patient with a too small remnant liver or resection proves technically too difficult. Disappearing liver metastases are described in only a few, mostly retrospective studies and the definition of DLM and used imaging modalities vary, along with the reported outcomes. Magnetic resonance imaging (MRI) has a higher sensitivity for detecting CRLM than contrast-enhanced computed tomography (CE-CT) [5], and it seems that gadoxetic acid (Gd-EOB-DTPA)-enhanced magnetic resonance imaging (EOB-MRI) increases the sensitivity even further [6, 7]. However, no universal approach exists for the assessment and management of DLM. Using recent advances in MRI technology combined with intraoperative ultrasound (IOUS), it has been possible to identify DLM with residual tumors with accuracies reaching as high as 83 and 90% [8, 9]. It is however unclear whether it is safe to leave in situ those CRLM that have become undetectable by pre- and intraoperative imaging after chemotherapy.

The primary aim of this systematic review was to summarize the existing knowledge on DLM and to investigate how well DLM correspond to a CR, i.e., resected DLM without signs of cancer on histopathology or DLM left in situ without recurrence. The secondary aim of this systematic review was to summarize outcomes in patients with DLM and to compare recurrence-free and overall survival in patients that undergo resection of DLM versus patients with DLM left in situ.

## Materials and methods

The study protocol was established and followed according to the PRISMA-P guidelines and checklist [10]. Study protocol can be found in Additional file 1 (supporting information). The review was registered in Prospero, the international prospective register of systematic reviews, on 27 September 2017, with registration number CRD42017070441 (see Additional file 1 for details).

### Search strategy, eligibility criteria, and data extraction

The literature search was developed with the aid of a university hospital librarian with a broad combination of medical subject headings and free-text words (Additional file 1). A literature search was performed in the PubMed and Embase databases through 6 October 2017 without restrictions in time. The following search combination was used: “((colorectal) AND ((disappear*) OR vanishing OR complete response OR missing)) AND ((“liver metastases” OR “liver metastasis”)).” The results yielded by the search were independently screened by two reviewers (DB and ES) using the web-based software platform Covidence. During the process of writing, PubMed was repeatedly searched until 30 October 2019 and reference lists of included studies were screened for additional studies of interest for this review. Studies eligible for inclusion were studies written in English reporting outcomes of DLM in adult patients undergoing surgery following chemotherapy for CRLM. Studies stating exclusive use of hepatic arterial infusion (HAI) or only biological agents were excluded as well as studies using fiducial placement. There was no restriction in study design, but case reports were excluded.

### Quality assessment

Quality assessment for included studies was done using the Newcastle-Ottawa Scale (NOS) for assessing quality of nonrandomized studies in meta-analyses. In-depth quality assessment of included studies may be found in Table S1 (supporting information).

### Definitions

There exists an inconsistent use of nomenclature on DLM making comparison of studies difficult. A DLM is a CRLM that becomes undetectable by any imaging following chemotherapy. Although a CRLM may disappear on any imaging, CE-CT is the most common imaging following chemotherapy. If the DLM becomes detectable by additional workup with MRI or IOUS (when CE-CT was the initial imaging method), it is no longer a DLM, but a residual CRLM that was not detected with the initial preoperative imaging. A remaining CRLM, in contrast to a residual CRLM, is a CRLM that never disappeared on preoperative imaging following chemotherapy. However, detectability by MRI or IOUS does not necessarily imply that this residual CRLM contains viable cancer cells on histopathology. Therefore, a residual CRLM that has turned fibrotic may still be defined as a complete pathologic response. Complicating matters additionally is the nomenclature used to describe the outcomes of DLM. According to the modified Response Evaluation Criteria in Solid Tumors (mRECIST), a CR is defined as the disappearance of any intratumoral arterial enhancement in all target or non-target lesions on imaging [11]. Some authors use the terms complete response/complete radiological response interchangeably when describing the disappearance of all liver metastases or as the complete disappearance of single metastasis [12, 13]. Others use the term CR as synonymous to complete pathological response (no viable cancer cells in resected DLM on histopathology) or complete clinical response (no recurrence of DLM left in situ during follow-up period). In this review, a CR is synonymous to a complete pathological response (CPR) or complete clinical response (CCR) and is not defined according to the response observed with imaging. See Table 1 for suggested nomenclature.

**Table 1:** Suggested nomenclature

Term	Explanation
Residual CRLM	DLM that became detectable by additional imaging
Remaining CRLM	CRLM that never disappeared after chemotherapy
Complete pathological response (CPR)	No viable cancer cells in resected DLM on histopathology
Complete clinical response (CCR)	No recurrence of DLM left in situ during follow-up
Complete response (CR)	CPR or CCR

*CRLM* colorectal liver metastases, *DLM* disappearing liver metastases, *CPR* complete pathological response, *CCR* complete clinical response, *CR* complete response

### Data analysis

After the inclusion of the eligible studies, data including number of patients in each diagnostic and treatment group, age, sex, and other demographic and cancer-related variables of the patients were extracted and compiled into tables. The definitions and outcomes were reported as stated in the original studies.

The aim of this study included conducting a meta-analysis by comparing recurrence-free survival and overall survival between patients and/or DLM that undergo resection versus no resection, as well as calculating differences between the imaging modalities in detecting DLM with CR. After the extraction of the data, it was found that there exists a significant clinical heterogeneity in the used diagnostic modalities, definitions of outcomes and reporting of the results (i.e., results reported by patient or by lesion) in the included studies. Therefore, conducting any meta-analysis was not feasible, and it was decided that the findings are reviewed in a systematic manner only. Therefore, the data analysis was purely descriptive, and the outcomes are presented as percentages.

## Results

**Fig. 1:**
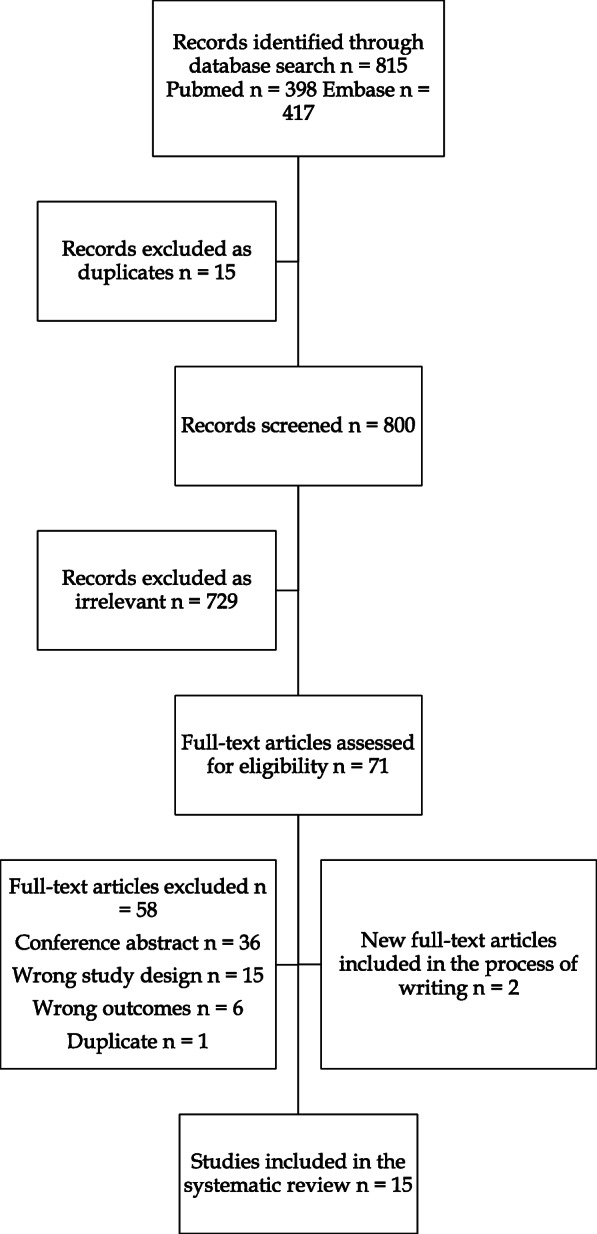
Selection of articles for review. Preferred Reporting Items for Systematic Reviews and Meta-Analyses (PRISMA) flow diagram

A total of 815 records were identified and screened in the PubMed and Embase databases. Details of the selection process are presented in the PRISMA-P flow chart, Fig. 1. Thirteen studies ultimately met eligibility criteria and were included in the review. Two additional studies were identified and included by regular searches in PubMed during the process of writing [8, 9].

### Baseline characteristics and treatments

Of the 15 included studies, 14 were retrospective cohort studies and one was a prospective cohort study [13]. Studies spanned the period of 1998–2015. Baseline characteristics of included studies are summarized in Table 2. In total, 3042 patients were included in this review with a mean age of 58.5% (range 30–83). Three studies included patients with initially unresectable disease only [14, 15, 18]. Because of the retrospective nature of included studies, imaging and neoadjuvant treatment varied. Chemotherapy was administered alone or in combination with targeted biological therapies. In five of the earlier studies, some of the patients received hepatic arterial infusion chemotherapy (HAI) [14–18]. For details, see Table 1. Three studies [14, 15, 18] reported outcomes of DLM in the view of the patient, i.e., per-patient analysis, and the remaining 12 according to individual DLM, i.e., per-lesion analysis.

**Table 2:** Baseline characteristics of included articles

Ref.	Period	Type of study	Neoadjuvant therapy	No. of patients, male (%)	Mean age, years (range)	Quality score (Newcastle-Ottawa)
Elias et al. [14]	1998–2002	Retrospective cohort	Systemic chemotherapy ± HAI	104 (n/a)	52 (40–63)	7
Benoist et al. [13]	1998–2004	Prospective cohort	Systemic chemotherapy	586 (68.4)	61 (n.s.)	7
Elias et al. [15]	1999–2004	Retrospective cohort	Systemic chemotherapy ± HAI	228 (37.5)	59 (41–70)	7
Auer et al. [16]	2000–2003	Retrospective cohort	Systemic chemotherapy ± HAI	435 (73)	53 (n.s.)	9
Tanaka et al. [17]	1998–2007	Retrospective cohort	Systemic chemotherapy ± HAI	63 (73.9)	62 (46–77)	9
Goèré et al. [18]	1999–2007	Retrospective cohort	Systemic chemotherapy ± HAI	523 (40.7)	52.4 (n.s.)	7
van Vledder et al. [19]	2000–2008	Retrospective cohort	Systemic chemotherapy ± biological agents	168 (n.s.)	n.s.	9
Ferrero et al. [20]	2004–2008	Retrospective cohort	Systemic chemotherapy	171 (75)	62 (n.s.)	7
Park et al. [21]	2008–2011	Retrospective cohort	Systemic chemotherapy ± biological agents	87 (77.1)	58.6 (n.s.)	7
Kim et al. [22]	2010–2012	Retrospective cohort	Systemic chemotherapy ± biological agents	137 (77.8)	60.3 (n.s.)	8
Arita et al. [23]	2011–2012	Retrospective cohort	Systemic chemotherapy ± biological agents	72 (69.4)	61 (42–81)	6
Owen et al. [24]	2008–2014	Retrospective cohort	Systemic chemotherapy ± biological agents	23 (n.s.)	53 (37–74)	7
Tani et al. [9]	2010–2014	Retrospective cohort	Systemic chemotherapy ± biological agents	82 (50)	57.5 (34–77)	6
Sturesson et al. [25]	2011–2014	Retrospective cohort	Systemic chemotherapy ± biological agents	179 (70)	68 (48–83)	7
Oba et al. [8]	2010–2015	Retrospective cohort	Systemic chemotherapy ± biological agents	184 (71)	59 (30–81)	6

*HAI* hepatic arterial infusion chemotherapy, *n/a* not applicable, *n.s.* not stated

### Patients, imaging, and management of DLM

Patients in the included studies had a total of 4742 CRLM. After chemotherapy, patients presented with 1561 DLM. The incidence of patients with 1 or more DLM ranged from 7 to 48% (median 19%). Patients had a median of 3.4 DLM per patient (range 0.4–5.6). Before chemotherapy, patients were evaluated with CE-CT in most cases, but in 4 studies, patients were additionally screened with MRI [14, 15, 24, 25], to a varying degree. In one study, MRI was the only imaging modality used before and after chemotherapy [22]. After chemotherapy, no standard imaging protocol was used preoperatively, to evaluate response to chemotherapy and identify DLM although CE-CT was used as a reference or baseline in most patients. In six studies [8, 9, 14, 15, 21, 23], all patients were evaluated with both CE-CT and MRI preoperatively. Types of imaging used before and after chemotherapy are presented in detail in Table 3. At surgery, IOUS was used in all patients except for one study [24] where IOUS was used selectively and in one study [22] where the use of IOUS was not stated. Contrast-enhanced IOUS was used in five studies [8, 9, 20, 23, 25]. Three studies [14, 15, 18] only included patients where DLM were left in situ and followed radiologically and consequently contained no data on histopathological outcomes. These studies were also the ones with a per-patient analysis. The remaining 12 studies reported outcomes of resected DLM as well as outcomes of DLM left in the remnant liver. Seven out of the 15 studies reported on recurrent DLM [9, 14, 16–18, 20, 21]. Most recurrences of DLM occurred within 2 years, and the median follow-up period was 29 months (range 12–55).

**Table 3:** Imaging for DLM

Ref.	Pre-chemotherapy staging (%)	Post-chemotherapy/preoperative staging (%)	IOUS
Elias et al. [14]	CE-CT (100) and MRI (100)	CE-CT (100) and MRI (100)	Yes
Benoist et al. [13]	CE-CT (100)	CE-CT (100)	Yes
Elias et al. [15]	CE-CT (100) and MRI (100)	CE-CT (100) and MRI (100)	Yes
Auer et al. [16]	CE-CT (100)	CE-CT (100)	Yes
Tanaka et al. [17]	CE-CT (100)	CE-CT and PET-CT (since 2002)	Yes
Goèré et al. [18]	CE-CT (100)	CE-CT (100)	Yes
van Vledder et al. [19]	CE-CT (100)	CE-CT (87) or CE-MRI (13)	Yes
Ferrero et al. [20]	CE-CT (100)	CE-CT and/or EOB-MRI	CE
Park et al. [21]	CE-CT (100)	CE-CT (100) and EOB-MRI (100)	Yes
Kim et al. [22]	EOB-MRI (100)	EOB-MRI (100)	n.s.
Arita et al. [23]	CE-CT (100)	CE-CT (100) and EOB-MRI (100)	IOUS and CE-IOUS
Owen et al. [24]	CE-CT (n.s.) or MRI (n.s.)	EOB-MRI (100)	Not universally performed
Tani et al. [9]	CE-CT (100)	CE-CT (100) and EOB-MRI (100)	CE
Sturesson et al. [25]	CE-CT (100) and additional EOB-MRI (62)	CE-CT (48), EOB-MRI (21), CE-CT and EOB-MRI (31)	IOUS and CE-IOUS
Oba et al. [8]	CE-CT (100)	CE-CT (100) and EOB-MRI (100)	CE

*CE-CT* contrast-enhanced computed tomography, *PET-CT* positron emission tomography, *n.s.* not stated, *MRI* magnetic resonance imaging, *EOB-MRI* gadoxetic acid-enhanced magnetic resonance imaging, *IOUS* intraoperative ultrasound, *CE-IOUS* contrast-enhanced intraoperative ultrasound

### Correlation of DLM to CR and imaging accuracies

Without stratifying outcomes according to chemotherapy and imaging methods used to identify DLM, this systematic review showed that a DLM signified a CR in 17–85% (median 54.5%).

With the use of IOUS and intraoperative palpation of the liver to rule out residual CRLM, the probability that the remaining DLM represented a CR increased significantly, compared to DLM that could be identified as residual CRLM. If the CRLM had disappeared on preoperative imaging and remained undetectable upon intraoperative examination with palpation and IOUS (with or without contrast), these DLM represented a CR in 24–96% (median 77.5%). See Table 4 and Table S2 (supporting information) for details.

**Table 4:** Complete response of DLM

Ref.	Patients with DLM (%)	Initial CRLM	DLM	DLM/patient	CPR/resected DLM	CCR/DLM left in situ	Time to recurrence	Median follow-up (months)	DLM with CR	DLM with CR + IOUS
Elias et al. [14]	15 (14)	n.s.	n.s.	n/a	n/a	8/11 **patients**	5, 5, 8 months	31	n/a	n/a
Benoist et al. [13]	38 (7)	183	66	1.7	3/15	8/31	n.s.	12	17%	24%
Elias et al. [15]	16 (7)	134	69	4.3	n/a	10/16 **patients**	n.s.	50	n/a	n/a
Auer et al. [16]	39 (9)	166	118	3	44/68	31/50	Mean 21 months	41	64%	65%
Tanaka et al. [17]	23 (37)	472	86	3.7	6/17	16/27	Median 14 months	44	69%	80%
Goèré et al. [18]	27 (n/a)	523	96	3.6	n/a	18/27	Median 14 months	55	n/a	n/a
van Vledder et al. [19]	40 (24)	n.s	127	3.2	26/67	24/45	n.s.	20	45%	54%
Ferrero et al. [20]	33 (19)	624	67	2	22/57	4/10	Within 12 months	n.s.	39%	64%
Park et al. [21]	87 (n/a)	393	CT: 203, MRI: 55	0.6 (MRI)	CT: 47/168, MRI: 28/39	CT: 24/35, MRI: 15/16	Within 12 months	12	CT: 35%, MRI: 78%	CT: 69%, MRI: 94%
Kim et al. [22]	43 (31)	289	168	3.9	8/8	128/150	n.s.	22	85%	n/a
Arita et al. [23]	11 (15)	234	32	0.4	10/37	4/7	n.s.	n.s.	41%	IOUS: 46%, CE-IOUS: 75%
Owen et al. [24]	11 (48)	200	77	7	10/36	20/41	n.s.	46	40%	n/a
Tani et al. [9]	20 (24)	619	111	5.6	CT: 54/78, MRI: 24/29	CT: 11/33, MRI: 16/18	Median 8 months	27	CT: 59%, +MRI: 85%	86%
Sturesson et al. [25]	29 (16)	141	66	2.3	24/56	3/4	n.s.	n.s.	45%	96%
Oba et al. [8]	59 (32)	764	275	4.7	103/233	36/42	n.s.	27	CT: 51%, +MRI: 65%	92%

*DLM* disappearing liver metastases, *CRLM* colorectal liver metastases, *CPR* complete pathological response, *CCR* complete clinical response, *CR* complete response, *n.s.* not stated, *n/a* not applicable, *IOUS* intraoperative ultrasound, *CE-CT* contrast-enhanced computed tomography, *MRI* magnetic resonance imaging, *EOB-MRI* gadoxetic acid-enhanced magnetic resonance imaging, *CE-IOUS* contrast-enhanced intraoperative ultrasound

According to preoperative imaging, a DLM on CE-CT was equal to a CR in 17–69% (median 51%) and 40–85% (median 78%) according to MRI. There were 3 studies specifically comparing CE-CT with EOB-MRI, and all of them showed that EOB-MRI was significantly more superior in detecting the DLM with CR [8, 9, 21]. In 4 studies where DLM on preoperative CE-CT were additionally evaluated with MRI and IOUS [8, 9, 21, 23], these remaining undetectable DLM were equal to a CR in 75–94% (median 89%). In 3 of these studies, addition of IOUS to MRI significantly improved detection of the DLM with CR [8, 21, 23] while in one the benefit was negligible [9]. Regarding detectability of CRLM, in the study by Tani et al. and Oba et al. [8, 9], all the remaining CRLM that were identified by preoperative CE-CT were also identified by MRI and IOUS. In the study by Tani et al. [9], 38.7% of DLM on preoperative CE-CT could be detected by both MRI and IOUS as residual CRLM. Additionally, 18.9% and 17.1% of DLM on CE-CT were detected exclusively by MRI or CE-IOUS, respectively. In the study by Oba et al. [8], 25% of DLM on preoperative CE-CT were detected by both MRI and IOUS, but in this study, only 1% of the DLM were detected exclusively by MRI and 34% were identified by IOUS alone.

Comparing detectability of residual CRLM with IOUS and CE-IOUS, in the study by Sturesson et al. [25], IOUS without contrast was able to detect 16 (24%) of the DLM from preoperative imaging as residual CRLM. Addition of contrast resulted in the detection of only 1 (26%) more residual CRLM. Similar results were obtained in the study by Ferrero et al. [20] with no difference in detection of residual CRLM with use of CE-IOUS. By contrast, in the study by Arita et al. [23], IOUS was able to detect 4 (13%) of the DLM on preoperative imaging and CE-IOUS were able to detect an additional 12 (50%). The sensitivity of IOUS to detect residual CRLM was 89% in the study by Arita et al. [23]. Corresponding sensitivity for CE-IOUS was 100%, 73%, and 93% in the studies by Arita et al., Tani et al., and Oba et al. [8, 9, 23], respectively.

### Patient outcomes

Outcomes in patients with DLM were reported in five studies [14, 17–19, 24], see Table 5 for details. A comparison of outcomes in patients with resected DLM vs. patients with DLM left in situ was available in three studies. Two of these studies compared data on overall- and disease-free survival [17, 19], and one study compared data on disease-free survival only [24]. There were no statistically significant differences in overall survival between patients with resected DLM and those without resection in two of the studies, while in the study by van Vledder et al. (*n* = 168), the patients with resected DLM had lower incidence of intrahepatic reoccurrence (69 and 35% 1- and 3-year disease-free survival vs. 40 and 16%).

**Table 5:** Patient survival

Ref.	Resection	Left in situ	Resection vs. no resection
Elias et al. [15]	n/a	3 years OS = 94% 3 years DFS = 64%	n/a
van Vledder et al. [19]	1, 3, and 5 years OS = 93, 59, and 38% 1 and 3 years DFS = 69 and 35%	1, 3, and 5 years OS = 94, 64, and 64% 1 and 3 years DFS = 40 and 16%	No statistically significant difference in OS but significant difference DFS
Tanaka et al. [17]	Median OS = 53 months Median DFS = 22 months	Median OS = 63 months Median DFS = 16 months	No statistically significant difference
Goèré et al. [18]	n/a	3 and 5 years OS = 87 and 80% 3 and 5 years DFS = 23 and 23%	n/a
Owen et al. [24]	Median DFS = 483 days	Median DFS = 360 days	No statistically significant difference

*n/a* not applicable, *OS* overall survival, *DFS* disease-free survival

## Discussion

This review shows that combining high-resolution EOB-MRI with CE-IOUS to evaluate DLM on preoperative CE-CT can identify DLM with CR with high probability. Whether leaving a DLM with CR in situ will result in different outcomes compared to resection could not be evaluated based on current evidence.

Due to heterogeneity in the definitions of DLM and data reported, a meta-analysis comparing diagnostic accuracy or outcomes in patients with DLM proved unfeasible. Studies exclusively treating patients with HAI were excluded because the numerous combinations of chemotherapy regimens have the capacity to confound the interpretation of the results from different studies. We selected studies with administration of standard chemotherapy regimens. HAI is not performed in many centers and its use is debated. However, several definitions concerning DLM were discussed in-depth in this review, with the aim to assist in reaching a standard nomenclature for future studies to decrease heterogeneity in the reporting of data on DLM. When deciding how to best manage DLM, a CR to either target or non-target lesions according to the radiologist as described by the mRECIST criteria is of little value for surgeons in the management of DLM. In this review, a CR was defined as either a CPR or a CCR. Potential methodological problems with this approach must be addressed. With histopathological assessment it is possible for minimal lesions to hide between the microscopic slides [17]. This may result in an overestimation of the CR rates with a CPR being one of two ways to conclude a CR. Because of this, Sturesson et al. [25] suggest CCR as a more reliable measure for CR. On the other hand, post-operative chemotherapy and/or liver ablation can potentially influence the CCR rate as it may result in lower recurrence rate [9]. Additionally, a median follow-up period of 29 months could imply that some of the patients did not have a sufficiently long-time span for the DLM to reoccur, although most DLM recurrences occurred within 2 years [12]. The incidence of DLM varied widely in this review (7–48%). Heterogeneity in types of imaging used before and after chemotherapy accounts for some of this variation. MRI, particularly with liver-specific contrast agent, is more sensitive in detecting small CRLM compared to CT [24]. Since smaller lesions are probably more likely to disappear following chemotherapy [19], it can be speculated that patients evaluated with EOB-MRI before chemotherapy will present more frequently with DLM. The latter statement is supported by the incidence of DLM reaching as high as 48% and 31% in the studies by Owen et al and Kim et al. [22, 24]. Chemotherapy with HAI has been proposed as another explanation for high incidence of DLM. In the study by Tanaka et al. [17] where as many as 68% patients were treated with HAI, 37% patients presented with one or more DLM and none of the patients were evaluated with MRI before chemotherapy, which hypothetically, could have accounted for this high incidence [12]. However, in the other three studies where patients were similarly treated with HAI, the incidence of DLM was lower, ranging from 7 to 14% [14–16, 18]. High incidences of DLM may also be attributed to incremental advances in chemotherapy and use of targeted biological therapies, as the incidence of DLM, with two exceptions [23, 25], tends to increase over time [24].

This review shows that the disappearance of CRLM on CE-CT is not equivalent to a cure, with approximately half of the DLM on preoperative CE-CT showing viable cancer cells on histopathology or reoccurring within the follow-up period. However, EOB-MRI performed before and after chemotherapy shows promise, with the study by Kim et al. [22] reporting CR rates as high as 85%. Meanwhile, a similar study by Owen et al. [24] using EOB-MRI before and after chemotherapy suggested a CR rate of 39%, which is considerably lower compared to the former study. Previously, discrepancies in CR rates could be argued to mainly be attributed to the use of HAI. However, since 2008, none of the included studies treated patients with HAI [22]. There are however differences between these studies: the former used MRI with superior resolution and only enrolled patients in whom EOB-MRI had been performed before and after chemotherapy [22]. In the study by Oba et al. [8], EOB-MRI failed to detect approximately half of the DLM with viable cancer. However, when EOB-MRI was combined with CE-IOUS to rule out DLM with residual cancer the CR rate of the remaining DLM increased to 92% [8]. Compared to Oba et al. [8], EOB-MRI seemed to be more accurate in identifying DLM without viable cancer in the study by Tani et al. [9], with a negative predictive value of 86% [9].

Regarding detectability of remaining CRLM, preoperative CE-CT seems of lower value, as all the remaining CRLM on preoperative CE-CT were also identified by EOB-MRI and CE-IOUS [8, 9]. CE-IOUS had higher sensitivity for detecting DLM as residual CRLM compared to IOUS without contrast in the study by Arita et al. [23]. Use of CE-IOUS also resulted in higher detection rates in the studies by Arita et al. and Sturesson et al. [23, 25].

In a review from 2016, a local recurrence rate of 33–74% was seen if the area of the DLM was left without resection [12]. However, many of the studies in this review did not use CE-IOUS systematically, and only few patients in that review were additionally evaluated with EOB-MRI.

There is a general lack of studies comparing resection vs. observation in relation to survival and recurrence, which is arguably the most important variable when considering how to manage patients with DLM. Based on the scarce data available on patient outcomes in this review, it is not possible to draw any firm conclusions.

However, from a per-lesion perspective, this review shows that when CRLM disappear on CE-CT and remain disappeared on additional workup with EOB-MRI, these DLM represent a CR with higher probability. With the additional use of IOUS, especially CE-IOUS, to rule out DLM with residual CRLM, the probability increases even further. In conclusion, a combination of all three imaging modalities shows promising results in accurately identifying DLM with CR. This suggests that leaving DLM in situ can be an alternative to surgical resection in these carefully selected DLM.

There are additional clinical and research implications to this review: (1) Heterogeneity of future studies on DLM could be avoided by adhering to a more uniform nomenclature. (2) Future studies should evaluate DLM in a standardized fashion with all three imaging modalities which would allow for meta-analysis of accuracy of different radiological modalities. Finally, this review calls for a prospective study comparing resection vs. no resection in patients with DLM.

## Supplementary information

**Additional file 1:** Appendix S1 Protocol and search terms

**Additional file 2:** Table S1 Quality assessment of cohort studies

**Additional file 3:** Table S2 Intra-operative decision-making, management and outcomes of DLM with calculation of response rates

## Data Availability

Documents with data supporting the results presented in the review are listed below, under “Supporting information.” The documents are available from the corresponding author on request.
