# Supplementary material for: Imaging in disappearing colorectal liver metastases and their accuracy: a systematic review
Source: World J Surg Oncol. 2020 Oct 8;18:264. doi: 10.1186/s12957-020-02037-w (PMC7545848; doi:10.1186/s12957-020-02037-w)
Supplement: Supplementary file 1 — Additional file 1: Appendix S1 Protocol and search terms [file 12957_2020_2037_MOESM1_ESM.docx]

**Appendix S1**

**Protocol**

**Title**

**1a Identification**

Management of disappearing colorectal liver metastases and patient survival: protocol for systemic a review

**1b Update**

This is not an update for a previous review

**2 Registration**

In accordance with the guidelines, our systematic review protocol was registered with the International Prospective Register of Systematic Reviews (PROSPERO) on the 19th of July 2017.

**Authors**

**3a Contact**

Darius Barimani, M.D., Resident, Upper Gastrointestinal Surgery, Karolinska University Hospital

[darius.barimani@yahoo.se](mailto:darius.barimani@yahoo.se)

Ernesto Sparrelid, M.D., Ph.D., Attending physician, Upper Gastrointestinal Surgery, Karolinska University Hospital

[ernesto.sparrelid@karolinska.se](mailto:ernesto.sparrelid@karolinska.se)

Adress:

Gastrocentrum K53, Karolinska Universitetssjukhuset, 141 86 Huddinge

Affiliations:

Department of Clinical Science Intervention and Technology (CLINTEC), H9, Karolinska Institutet

**3b Contributions**

Will be completed with the review.

**4 Amendments**

No amendments as of yet. In case of future amendments they will be listed here.

Support

**5a Sources**

No sources of financial support.

**5b Sponsor**

This review is not sponsored.

**5c Role of sponsor or funder**

We have no financial support from any corporation or institution in writing this review but throughout the process of writing we will take help from the Department of Clinical Science Intervention and Technology (CLINTEC) at Karolinska Institutet in the form of expert advice from senior researchers and colleagues as well as assistance from their biostatisticians regarding the statistical content.

**Introduction**

**6 Rationale**

Approximately 30% of patients with colorectal cancer will at some point develop liver metastases. In the neoadjuvant era with modern cytotoxic agents some of these liver metastases disappear on presurgical staging. The rate of disappearing liver metastases (DLMs) depend on various factors such as; type of chemotherapy used and way of administration, patient- and tumor characteristics and so forth. However the ability to predict a true complete response of DLMs (no recurrence on follow up imaging or viable disease in resected specimen) has been shown to rely most heavily on type of imaging used where so far contrast enhanced MRI seems to be far superior to all other imaging modalities.

DLMs are a rather new phenomenon and only a small number of mainly retrospective studies have been published. With this review we hope to bring new light to the subject by taking into account newly published data on DLMs with contrast enhanced MRI and combine it with a meta analysis on overall patient survival.

Hitherto the philosophy towards DLMs has been that of an aggressive surgical approach were surgeons will try to resect all the areas of disappearing lesions. Resecting all original sites is probably the wisest but sometimes these sites can’t be localized and sometimes resecting them will leave a too small FLR. If survival can be proven to be the same in patients where DLMs were left in situ and follow up imaging at regular intervals turns out to be a safe alternative it could leave the surgeon with more options and save him or her from intraoperative angst.

**7 Objectives**

The aim of this review is to make qualitative and quantitative analysis of all available data on DLMs in order to answer the following questions:

1. What are the capabilities of different imaging modalities in predicting a true complete response?
2. What is the overall patient survival in patients were all DLMs were resected compared to were DLMs left in situ?
3. Is there support in the research for leaving the sites of DLMs?

**8 Eligibility criteria**

Studies will be selected according to criteria outlined below:

Study design:

No restriction in study design

Case studies will be excluded

Participants:

Inclusion of all studies reporting outcomes on patients with the following characteristics:

- Adult patients with colorectal cancer with imaging before and after chemotherapy and at least one reported DLM post chemotherapy
- Resection of at least one DLM
- Conventional chemotherapeutic agents including biological agents
- Studies reporting both pathologic and complete radiological response

Exclusion:

- Presence of extrahepatic metastasis
- Case reports
- Studies only using HAI (hepatic arterial infusion chemotherapy)
- No DLMs left in situ

Interventions

Surgical resection of DLM?

Comparators

No resection of DLM?

Outcomes

???

**9 Information sources**

Our search strategy will consist of using a broad combination of medical text words in order to pick up all relevant articles pertaining to the subject of DLM from article titles or abstracts. Searches will be conducted on Pubmed and Embase from the year 1990 and onwards. In addition to database searches we will also look for additional articles in the reference list of included studies.

**10 Search strategy**

Combination of medical text word searches listed below created with the aid of clinical librarian at Karolinska University hospital. The search will be narrowed to english language articles only from year 1990 and onwards.

PROSPERO and ClinicalTrials.gov will also be searched for reviews or other articles in the making.

Draft of Pubmed and Embase search strategies will be included in appendix 1.

**11a Data management**

Keynote will be used for purposes of overview, reference and extracting fulltext articles.

**11b Selection process**

The review authors will independently screen abstracts yielded by the search and obtain fulltext of studies meeting eligibility criteria for further analysis as well as identifying additional articles through references of articles found by the search.

Review authors will then met and compare results discussing and resolving eventual differences in order to assure the inclusion of all relevant articles.

**11c Data collection process**

Reviewers will independently read through abstracts yielded by the search. Abstracts of interest will be identified and their full texts will be obtained. We will use Covidence.org for this purpose.

**12 Data items**

???

**13 Outcomes and prioritization**

Main outcomes:

Number of DLM by imaging modality and article

Number of DLM showing complete clinical response and complete pathological response

Positive predictive value for absence of tumor imaging modality and article.

Rationale: What should be the standard imaging modality of choice for presurgical staging and for treatment and follow-up of DLM in general?

5 year patient survival where all DLM sites resected

5 year patient survival where DLM sites ablated or left in situ

Rationale: Most authors recommend resection of all DLM sites. This is not always possible or preferable e.g. small remnant liver mass. Resection of all sites does not seem to have a statistical significant effect on overall patient survival why optional strategies could be adapted (clinical surveillance, ablation etc).

Secondary/additional outcomes:

Median time to recurrence of DLM

Rationale: Optimal timing of clinical follow up.

To discover or assesing certain factors (e.g. patient characteristics, chemotherapy) as especially significant in predicting a true complete response or patient survival.

**14 Risk of bias in individual articles**

Cochrane Collaboration tool for assessing the risk of bias. Behöver vi använda en sån?

**Datasynthesis**

15a Describe criteria under which study data will be quantitatively synthesised

???

15b If data are appropriate for quantitative synthesis, describe planned summary measures, methods of handling data and methods of combining data from studies, including any planned exploration of consistency (such as I 2, Kendall’s τ)

???

15c Describe any proposed additional analyses (such as sensitivity or subgroup analyses, meta-regression)

???

15d If quantitative synthesis is not appropriate, describe the type of summary planned

???

16 Meta-bias(es) Specify any planned assessment of meta-bias(es) (such as publication bias across studies, selective reporting within studies)

???

17 Confidence in cumulative evidence. Describe how the strength of the body of evidence will be assessed (such as GRADE)

???

**Prospero**

Date of submission: 18 Aug 2017

Date of publication on Prospero database: 27 Sep 2017

Registration no.: CRD42017070441

**Search terms**

Embase:

colorectal AND (disappear* OR vanishing) AND ('liver metastases' OR 'liver metastasis'/exp OR 'live metastasis')

AND [embase]/lim NOT [medline]/lim

Pubmed:

((colorectal) AND ((disappear*) OR vanishing OR complete response OR missing)) AND (("liver metastases" OR "liver metastasis"))
