# Supplementary material for: Imaging in disappearing colorectal liver metastases and their accuracy: a systematic review
Source: World J Surg Oncol. 2020 Oct 8;18:264. doi: 10.1186/s12957-020-02037-w (PMC7545848; doi:10.1186/s12957-020-02037-w)
Supplement: Supplementary file 2 — Additional file 2: Table S1 Quality assessment of cohort studies [file 12957_2020_2037_MOESM2_ESM.docx]

Quality assessment of cohort studies

| Cohort Star Template | | | |
| --- | --- | --- | --- |
| Article | Selection | Comparability | Outcome |
| Elias, 2004 | 4 | 0 | 3 |
| Benoist, 2006 | 4 | 0 | 3 |
| Elias, 2007 | 4 | 0 | 3 |
| Auer, 2010 | 4 | 2 | 3 |
| Tanaka, 2009 | 4 | 2 | 3 |
| Goèré, 2011 | 4 | 0 | 3 |
| Van Vledder, 2010 | 4 | 2 | 3 |
| Ferrero, 2012 | 4 | 2 | 1 |
| Park, 2017 | 4 | 2 | 1 |
| Kim, 2017 | 4 | 1 | 3 |
| Arita, 2014 | 4 | 0 | 2 |
| Owen, 2015 | 4 | 0 | 3 |
| Tani, 2018 | 4 | 0 | 2 |
| Sturesson, 2015 | 4 | 1 | 2 |
| Oba, 2018 | 4 | 0 | 2 |
