# Supplementary material for: Imaging in disappearing colorectal liver metastases and their accuracy: a systematic review
Source: World J Surg Oncol. 2020 Oct 8;18:264. doi: 10.1186/s12957-020-02037-w (PMC7545848; doi:10.1186/s12957-020-02037-w)
Supplement: Supplementary file 3 — Additional file 3: Table S2 Intra-operative decision-making, management and outcomes of DLM with calculation of response rates [file 12957_2020_2037_MOESM3_ESM.docx]

**Intra-operative decision-making and evolution of DLMs**

| Ref. | IOUS | Intraoperative management and evolution | % of DLM on presurgical imaging with CR |
| --- | --- | --- | --- |
| Elias 2004 | Yes | N/a | N/a |
| Benoist 2006 | Yes |  | 11/66=16,7%  +IOUS:  11/46=23,9% |
| Elias 2007 | Yes | N/a | N/a |
| Auer 2010 | Yes | DLM was only left in situ in patients in whom resection of DLMs would result in an insufficient hepatic remnant. | 75/118=63,6%  +IOUS  70/107=65,4% |
| Tanaka 2009 | Yes |  | 50/72=69,4%  +IOUS  44/55=80,0% |
| Goèré 2011 | Yes | N/a | N/a |
| Van Vledder 2010 | Yes |  | 50/112=44,6%  +IOUS  31/57=54,4% |
| Ferrero 2012 | CE |  | 26/67=38,8%  +IOUS  14/22=63,6% |
| Park 2017 | Yes. Use of contrast n.s. |  | CT  71/203=35,0%  +IOUS  24/35=68,6%  MRI  43/55=78,2%  +IOUS  15/16=94,0% |
| Kim 2017 | n.s. | Out of 168 DLM, 8 were resected and showed no viable cancer cells. Additionally, for 10 DLM the patients received chemotherapy without cessation during the follow-up period because of presence of other CRLM which were considered viable. None of the 150 DLM were followed up with local treatment or chemotherapy. | 128/150=85,3%  +IOUS  n/a |
| Arita 2014 | Yes. All patients examined with and without contrast. |  | 13/32=40,6%  +IOUS  13/28=46,4%  +CE-IOUS  12/16=75,0% |
| Owen 2015 | Not universally performed | N/a | 30/77=38,9% |
| Tani, 2018 | CE | 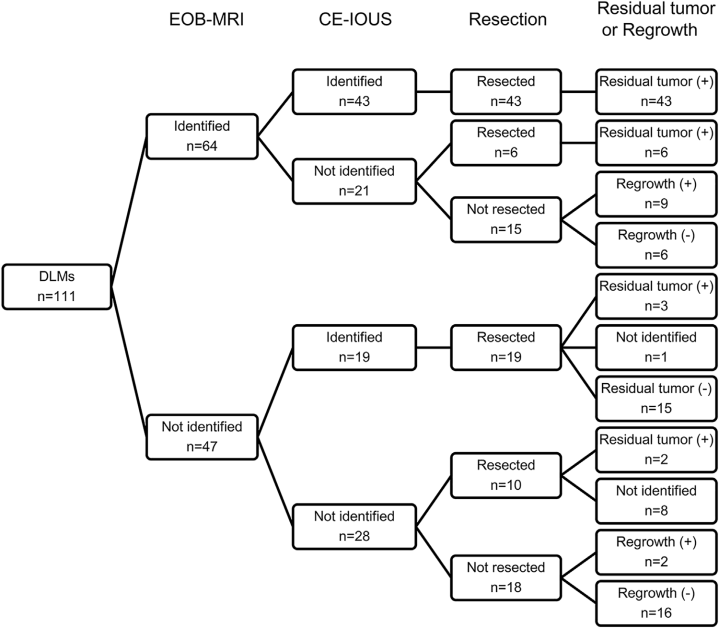 | 65/111=59.0%  +MRI  40/47=85.1%  +CE-IOUS=  24/28=85.7% |
| Sturesson, 2015 | With and without CE |  | 27/60=45.0%  +CE-IOUS  23/24=95.8% |
| Oba, 2018 | CE |  | 139/275=50.5%  +MRI  133/204=65.2%  +CE-IOUS  101/110=91.8% |
| IOUS: Intra-operative ultrasound; DLM: Disappearing liver metastases; CR: Complete response; N/a: Not applicable; CE-IOUS: Contrast-enhanced intra-operative ultrasound | | | |
